# Fatal Case of Visceral Disease, with Cerebral Symptoms—Post Mortem Examination

**Published:** 1845-03

**Authors:** J. Stickel

**Affiliations:** Galena, Ill.


*Fatal Case of Visceral Disease, with Cerebral Symptoms—Post Mortem Examination.* By J. STICKEL, M.D., of Galena, Ills.

GALENA, Feb. 14, 1845.

D. BRAINARD, M. D.:

SIR,—The following case, which recently occurred in my practice, may possess some interest, and on that account I concluded to report it to you.

On the 28th January, John Miller, a Carpenter, aged about 35 years, called at my office, and stated to me that he was unwell, and asked me to take charge of his case. On inquiry, I found he had been unwell for two years; had an attack of remittent fever in the summer; suffered a great deal from delirium at that time; had since that time frequently been almost blind, from what he called weakness of his eyes. On examination, I found him laboring under disease of the liver, which appeared to be enlarged and hard, and tender on pressure over the region of it. I applied a blister over the region of the liver, and his bowels being costive, gave him a dose of calomel and ipecac, which did not operate. The next day I followed it with a dose of ol. Ricini, and procured free bilious discharges. The calomel produced a smart ptyalism, and he continued to discharge bile of a brownish color, by emesis and by stool, in large quantities. There was also continued hiccough. To allay the latter, I gave laudanum, ether, and other antispasmodics, in large doses, but they had no effect. The discharges and hiccough continued, accompanied by a degree of delirium—he would answer questions correctly, but talked incoherently—until the evening of the 7th February, when he died. During the whole of his sickness there was no febrile action, the pulse being low, the pupils of his eyes contracted, and the surface cool.

*Post Mortem Examination* 12 *hours after Death.*—On opening the cranium, I found extensive effusion had taken place. The dura mater and pia mater were adherent to each other, over the major part of both lobes of the brain. The falx cerebri was partly decomposed, and the ventricles were filled with water, and the whole substance of the brain very much softened. On opening the chest and abdomen, I found the lungs very dark colored, the right lobe adherent to the pleura, a considerable quantity of lymph in the air cells, but no tubercles. The right ventricle of the heart was hypertrophied, and the left very much thickened. The liver was very much enlarged, very dark-colored, and the whole upper surface of the right lobe was firmly adherent to the diaphragm. The stomach and intestines appeared healthy. I suppose the hiccough, upon which all remedies seemed to have no effect, was caused by the adhesion of the liver to the diaphragm.

Yours, respectfully,

J. STICKEL.

